# Spatiotemporal Variations in Nectar Robbing and Its Effects on Reproduction in *Salvia castanea* Diels (Lamiaceae)

**DOI:** 10.3390/plants14152266

**Published:** 2025-07-23

**Authors:** Han-Wen Xiao, Yan-Bo Huang

**Affiliations:** 1 Eastern China Conservation Centre for Wild Endangered Plant Resources, Shanghai Chenshan Botanical Garden, Shanghai 201602, China; 2 College of Landscape Architecture and Art, Fujian Agriculture and Forestry University, Fuzhou 350002, China; 3 College of Landscape Architecture, Nanjing Forestry University, Nanjing 210037, China

**Keywords:** floral longevity, nectar availability, nectar robbing, pollen deposition, reproductive success, *Salvia*

## Abstract

Nectar robbing typically reduces nectar availability to pollinators, damages flower structure, and/or induces secondary robbing. Consequently, it may reduce pollen deposition and seed set, increase pollination efficiency and outcrossing, and/or not affect reproduction in some species. However, spatiotemporal variations in nectar robbing and their effects on plant reproduction have received little attention. In this study, we assessed the effects of nectar robbing on floral visits, seed set, nectar volume and concentration, and flower longevity in two populations of *Salvia castanea* Diels (Lamiaceae) in the Himalayan region of Southwestern China in 2014–2020. We also examined whether one or a few visits by pollinators can result in the stigma receiving sufficient pollen to fertilize all ovules of *S. castanea*. We found that significant differences in the nectar robbing rate did not affect seed set in any of the years for either population of *S. castanea*. In the robbed and unrobbed flowers, nectar was consistently replenished every night at higher concentrations. Bagging, nectar robbing, and sufficient pollination did not affect flower longevity. *Salvia castanea* required only 5–10 pollen grains to achieve the maximum seed set. However, pollinators depositing more than 10 pollen grains after a single visit ensured a high seed set of >80%. Our results suggest that nectar availability, floral longevity maintenance, and sufficient pollen deposition mitigate the effects of nectar robbing on the reproductive success of *S. castanea*. These results are expected to further our understanding of plant–animal interactions and the ecological consequences of nectar robbing.

## 1. Introduction

In the complex network of ecological relationships between animals and plants, nectar robbing, as a specialized interspecific interaction, has long attracted extensive attention from ecologists [1,2,3,4,5,6,7,8,9,10]. When exploring the effects of nectar robbing on plant reproduction, it is critical to accurately distinguish between effective and ineffective pollinators. Effective pollinators are those animals that are able to successfully transfer pollen to the stigma during flower visits to achieve pollination for plant reproduction [11,12]. These pollinators usually have morphological features and behavioral patterns that are compatible with the structure of the flower [12,13]. When they visit flowers, their bodies are usually in contact with the stamens and pistils, allowing pollen to attach to the body and transfer to the pistil of another flower without difficulty [12,13]. In contrast, ineffective pollinators obtain only nectar or pollen reward during flower visits and are unable to effectively complete pollen transfer to facilitate fertilization and seed production [11]. In pollination mutualism, cheaters can be categorized as robbers or thieves based on whether they make holes during flower visits or not [1]. Nectar robbing, the ineffective visitation of birds or insects that poke holes directly in flowers to obtain nectar without providing pollination services [1,2], is a widespread phenomenon that has been documented in more than 59 plant families of tubular and nectar spur flowers worldwide [3,4]. This behavior not only changes the reproductive strategies of plants but also profoundly affects the structure and function of ecosystems.

It has been shown that nectar robbing typically has different effects on plant reproduction and fitness, varying from negative to positive or neutral [5,6,7,8,9,14]. Robbers destroy flower structure by punching holes in the calyx or corolla tube, leading to a shortened flower lifespan or increased plant resource costs, which ultimately reduce seed set [6,15]. Indirectly, they may also change the foraging behavior of effective pollinators, reduce attraction to pollinators, or induce secondary robbing [16,17]. In addition, nectar robbing may indirectly benefit plant reproduction by reducing nectar availability to pollinators, forcing them to travel farther and visit more flowers to obtain nectar, thereby increasing pollination efficiency and outcrossing [8,18,19]. Some other studies have examined the effects of nectar robbing on maintenance neutral effects, including nectar availability, non-discrimination, and visitor prevalence, and the pollen saturation hypothesis [8,20,21,22]. However, despite the prevalence of nectar robbing, its spatial and temporal variation and effects on plant reproduction have received little attention and deserve further investigation.

The intensity of nectar robbing and its effects on plant reproduction vary over time and space [3,23,24]. For example, variation in the intensity of nectar robbing among populations and between years was observed in *Antirrhinum valentinum* Font Quer (Plantaginaceae), but there was no evidence that nectar robbing had an effect on its reproductive success [20]. In *Salvia gesneriflora* Lindl (Lamiaceae), there were substantial spatiotemporal variations in nectar robbing, with nectar availability in unrobbed flowers being approximately eight times that of robbed flowers, but there was no difference in their floral longevity [23]. Also, in non-pollen-limited, self-compatible, and few-ovule species, a single visit by a pollinator readily deposits the amount of pollen needed to achieve adequate fertilization [25]. In this case, spatiotemporal variation in nectar robbing may not negatively affect its reproductive success [22]. Undoubtedly, the effect of nectar robbing on plant fitness may exhibit greater complexity and variability when spatiotemporal variations in nectar robbing rate are considered. Thus, exploring the effects of spatiotemporal variations in nectar robbing on plant reproduction is critical to understanding plant–nectar robber relationships.

*Salvia* is the largest genus in the Lamiaceae, with approximately 1000 species distributed worldwide [26,27,28,29]. This genus has a staminal lever structure adapted for pollination by nectarivorous birds and bees [25,27,28,29,30,31,32,33,34,35,36]. Currently, a few studies have examined the effects of nectar robbing on the reproductive success of *Salvia* [8,9,23,37], providing a framework for further research on the effects of nectar robbing on the reproduction of *Salvia*.

In this study, we assessed the effects of nectar robbing on floral visits and seed sets in two populations of *S. castanea* in the Himalayan region of Southwestern China during 2014–2020. Temporal variations in nectar volume, concentration, and floral longevity were compared between robbed and unrobbed flowers. We examined the amount of pollen needed to achieve sufficient fertilization, the amount of pollen deposited, and the seed set during a single visit. The results of this study answer the following questions: (1) Does nectar robbing affect the reproductive success of *Salvia castanea*? (2) How does *S. castanea* maintain its reproductive success under the spatiotemporal variations in pollinators and nectar robbers? Our results provide evidence for a comprehensive understanding of the impact of plant–animal interactions on reproductive success and also add to the literature on studies related to the impact of nectar robbing on reproductive success in this genus.

## 2. Materials and Methods

### 2.1. Study Sites and Species

This study was conducted between July and October from 2014 to 2020 at Yulong Mountain (YL, 27°8′ N, 100°15′ E) and Mingyin Township (MY, 27°12′ N, 100°20′ E) in Lijiang City, Yunnan Province, China. The altitudes of the two sites were 3050 and 2750 m, with a straight-line distance of approximately 30 km. The two sites have southwest and Indian Ocean monsoon climates and a subtropical plateau dry hot river valley climate, respectively. The region has distinct wet and dry seasons, with the dry season from November to May and the rainy season from June to October. At the two locations, the average annual temperatures were 12.8 °C and 10.7 °C, and the average annual precipitation was 935 mm and 684.8 mm, respectively [27,28,38,39].

Sage has only four small ovules, and insects could easily deposit more than four pollen grains on the stigma during a single visit [13,25,28]. *S. castanea* is distributed in alpine meadows and forested open grasslands from the northern slopes of the eastern section of the Himalayas to the southwestern part of the Hengduan Mountains at an altitude of approximately 3000 m. This species is a perennial herb with a height of 30–65 cm, a flowering period from August to October, an average inflorescence number of four per plant, and nectar production at the base of the corolla tube [27,28]. Previous studies have found that large inter-annual variations in pollinator composition and behavior, and frequent nectar robbing have been reported in this species. In addition, this species has no pollen or pollinator limitations, exhibits a low degree of autonomous self-pollination, and has a mixed mating system [27,28].

### 2.2. Effect of Nectar Robbing on Floral Visits

Previous studies have documented the types and behaviors of pollinators and robbers of *S. castanea*. To document the spatiotemporal variations in pollinator and nectar robber visitation rates, and the effect of nectar robbing on visitation rates, we watched videos recorded by Xiao et al. [27,28] and Chang et al. [38] in the YL (2014–2016 and 2020) and MY (2019) populations, respectively. Pollinator and nectar robber visitation rates in the MY population were recorded using two HDR-CX510E Sony cameras (SONY Group Corp., Minato, Japan) from 8:00 to 18:00 daily from 24 to 30 August 2020. All of the above video recordings were taken using one camera per plant. The average number of flowers recorded per day per video is displayed in Table 1. We then recorded the visiting behavior of the insects in each of these videos; pollinators were defined as insects that touched stamens or stigmas while obtaining nectar [25], and robbers were defined as those that made holes in the base of the calyx or corolla tube to obtain nectar (or obtained nectar from existing holes, referred to as secondary robbers) and did not contribute to pollination [2,40]. We recorded the number of visits by all effective pollinators and nectar robbers on each plant per day. We expressed the effective pollinator and nectar robber visitation rates by dividing the number of effective pollinator and nectar robber visits by the total number of flower visits by all the insects. In addition, we calculated the average number of visits per flower per day for effective pollinators by dividing the total number of pollinator visits per day by the number of flowers observed per day. Furthermore, we explored the effect of robber visitation rate on effective pollinator visitation using Pearson correlation analysis.

### 2.3. Spatiotemporal Variations in Robbing Rate and Their Effects on Reproduction

To quantify the spatiotemporal variations in the robbing rate of *S. castanea* and their effects on seed set, we randomly labeled 26–30 plants per population and recorded the total number of flowers per plant, the number of flowers robbed, and seed sets in 2016, 2019, and 2020 in the YL population and in 2019 and 2020 in the MY population. We calculated the nectar robbing rate per plant as the ratio of the number of flowers with nectar robbing holes per plant to the total number of flowers on that plant.

### 2.4. Effects of Nectar Robbing on Nectar Availability and Flower Longevity

To detect nectar robbing on the availability of nectar volume and concentration to pollinators, we examined the nectar volume and concentration of robbed and unrobbed flowers from the YL population at 8:00–18:00 with 2 h intervals in 2020. A total of 180 flower buds were randomly selected (four flowers per plant) and bagged in nylon mesh bags. When all 120 of these flowers opened, the nylon bags were removed, allowing the flowers to be robbed; subsequently, we examined the nectar volume (20 flowers per time) of the robbed flowers using capillary tubes with a diameter of 0.5 mm and a height of 100 mm. Nectar concentration (20 flowers per time) was measured using a portable hand-held refractometer (LH-T50, Lohand Biological Group Corp., Hangzhou, China) with a 0–50% range and a 0.5% accuracy. The remaining 60 flowers were bagged consistently (10 flowers per time). We examined the nectar volume and concentration of the unrobbed flowers.

To test whether nectar robbing affects flower longevity and whether sufficient hand pollination shortens flower longevity, we randomly selected 100 flower buds from 10 plants (10 flowers per plant) of the YL population and covered them with nylon mesh bags. We defined the floral longevity of *Salvia* as the time from the slight opening of the upper and lower lips (0 h) to the complete wilting and withering of the entire flower, as described by Xiao et al. [25]. Flower longevity was recorded after the following five treatments (two flowers per plant per treatment) were applied at flowering (0 h): (1) bagged without any treatment, (2) robbed flowers, (3) flowers under natural conditions, and (4) flowers of geitonogamy, and (5) xenogamy.

### 2.5. Amount of Pollen Needed for Sufficient Fertilization, and Deposited Pollen Counts and Their Seed Set After Single Visit

We determined the amount of pollen needed for sufficient fertilization of *S. castanea*. Based on the fact that sage has four ovules per flower, 150 flowers were divided into five treatments (30 flowers per treatment): 1–3, 4, 5–10, 11–20, and >20 pollen grains were deposited per stigma, respectively, following the method described by Xiao et al. [25]. The pollinated flowers were immediately covered with nylon mesh bags, and the seed set for each flower was counted after 3–4 weeks.

To test the pollen saturation hypothesis in *S. castanea*, i.e., a single or a few visits by pollinators can result in the stigmas receiving enough pollen to fertilize all ovules, a total of 70 and 60 flower buds from the YL and MY populations, respectively, were randomly selected (two flowers per plant) and bagged in 2020. The bags were removed after flower opening to allow pollinators to visit, and the number of pollen grains deposited on the stigma was observed after a single visit of effective pollinators by taking 50 and 30 pistils from the YL and MY populations, respectively, under a portable electron microscope (STV-120m, Chiyoda-ku, Japan, Kenko). The remaining flowers (20 and 30 flowers) were bagged with nylon bags, and the seed set after a single visit was counted after 3–4 weeks.

### 2.6. Statistical Analysis

All statistical analyses were performed using IBM SPSS 22.0 (Chicago, IL, USA). All experimental data were analyzed using a generalized linear model (GLM) with a Gaussian distribution and a log link function. The likelihood ratio test (LRT) was used to compare the full model with the restricted model, and *p* values were calculated using an LRT χ^2^ distribution. The dependent variables were nectar robbing rate, seed set, floral visitation rate, nectar volume, concentration, flower longevity, and deposited pollen count, whereas the fixed factors were population, time, and treatment. Our Levene’s test for the above log-transformed data showed no significant difference in variance between the different groups (*p* > 0.05). Therefore, differences in the above between treatments or among populations were analyzed by a post hoc Tukey’s test. In this study, for percentages, the mean and its standard error (SEM) are calculated based on the least squares mean, and all other values are mean ± SE.

## 3. Results

### 3.1. Effect of Nectar Robbing on Floral Visits

**Figure 1:**
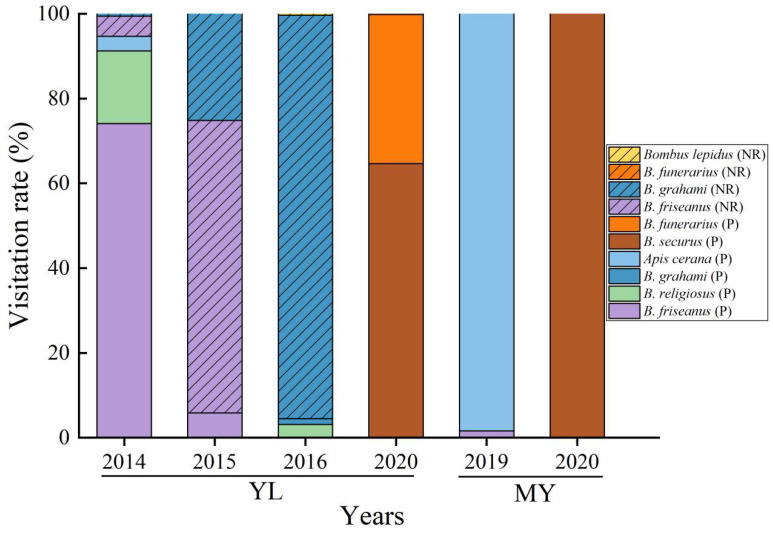
Visitation frequency of pollinators and robbers in both populations of *Salvia castanea* from 2014 to 2020. YL, Yulong Mountain; MY, Mingyin Town; NR, nectar robber; P, pollinator.

From 2014 to 2020, we recorded six effective pollinators, *Bombus friseanus*, *B. religiosus*, *B. grahami*, *B. securus*, *B. funerarius*, and *Apis cerana*, and four robbers, *B. friseanus*, *B. grahami*, *B. funerarius*, and *B. lepidus*, in YL populations. Among these, *B. friseanus*, *B. grahami*, and *B. funerarius* act as both pollinators and nectar robbers (Figure 1). Effective pollination and nectar robbing behaviors are shown in Figure S1. However, nectar robbing was not observed in the MY population (Figure 1). Interestingly, large inter-annual differences in pollinator composition and behavior were found between the two populations. The pollinator and nectar robber visitation rates ranged from 0.16% to 95.54% each year in the YL population (Table 1). Effective pollinator visit frequency analysis revealed significant differences in the average number of pollinator visits per flower per day in different years between the two populations (χ^2^ = 32.265, *df* = 5, *p* < 0.001, Table 1). In 2020, the average number of pollinator visits per flower per day was the highest among all years sampled in both populations, at 34.91 and 7.26, respectively. The average number of pollinator visits per flower per day was low, and no significant difference was found between the two populations in the other years (*p* = 0.057, Table 1). Further Pearson analyses showed that robber visitation rates were negatively associated with the average number of visits per flower per day by pollinators but were not significantly different (y = −0.1153x + 11.549, R^2^ = 0.1697, *p* = 0.417).

### 3.2. Spatiotemporal Variations in Robbing Rate and Their Effects on Reproduction

**Figure 2:**
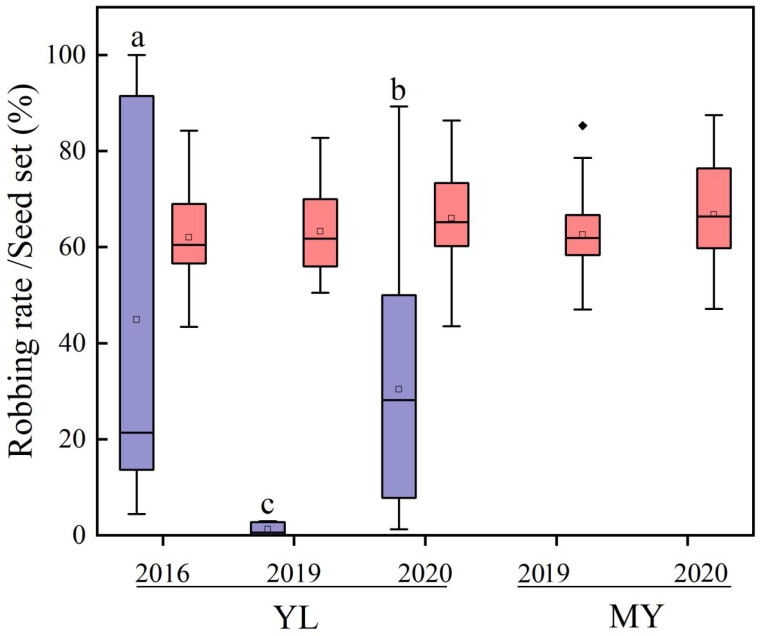
Nectar robbing rate (purple) and seed set (pink) in different years in both populations of *Salvia castanea*. Different lowercase letters indicate significant differences in nectar robbing rates. The boxes in the figure indicate the 25–75% data distribution range; the black horizontal line inside the box indicates the median; the hollow square inside the box indicates the mean; the upper and lower whisker lines indicate the maximum and minimum values, respectively; and the black solid rhombus outside the upper and lower whisker lines indicates the outliers (All of the following box plots are annotated to match here). YL, Yulong Mountain; MY, Mingyin Town.

Nectar robbing rates significantly varied between populations and years, with significant differences in average robbing rates of 44.93% ± 6.97%, 1.23% ± 0.24%, and 29.37% ± 4.38% in 2016, 2019, and 2020 (χ^2^ = 5.346, *df* = 4, *p* = 0.254; Figure 2), respectively, for the YL population. In contrast, there were no robbing holes in the MY population (Figure 2). However, no significant difference in seed set of almost 62% was found between populations and years (Figure 2).

### 3.3. Effects of Nectar Robbing on Nectar Availability and Flower Longevity

**Figure 3:**
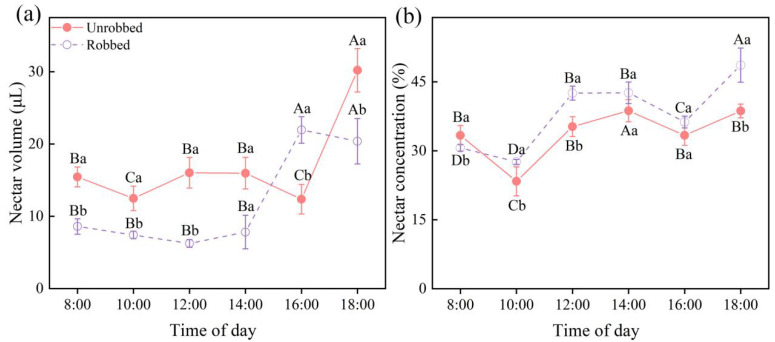
Temporal variation in nectar volume (**a**) and concentration (**b**) in robbed and unrobbed flowers. Different capital letters indicate significant differences in nectar volume and concentration between robbed and unrobbed flowers at different times of the day. Different lowercase letters indicate significant differences in nectar volume and concentration between robbed and unrobbed flowers at the same time.

The nectar volumes (robbed, χ^2^ = 11.720, *df* = 5, *p* = 0.039; unrobbed, χ^2^ = 123.505, *df* = 5, *p* < 0.001; Figure 3a) and concentrations (robbed, χ^2^ = 161.167, *df* = 5, *p* < 0.001; unrobbed, χ^2^ = 79.624, *df* = 5, *p* < 0.001; Figure 3b) of the robbed and unrobbed flowers significantly differed with time. The nectar volume of the unrobbed flowers rapidly replenished after 16:00, reaching 30 μL, whereas that of the robbed flowers was significantly lower than that of the unrobbed flowers before 14:00. The nectar volume of the robbed flowers rapidly replenished after 14:00 to the same level as the unrobbed flowers (Figure 3a). The nectar concentration in the unrobbed flowers was higher than that in the robbed flowers only at 8:00, and the nectar concentration in the robbed flowers was higher than that in the unrobbed flowers for almost all the other times (Figure 3b).

**Figure 4:**
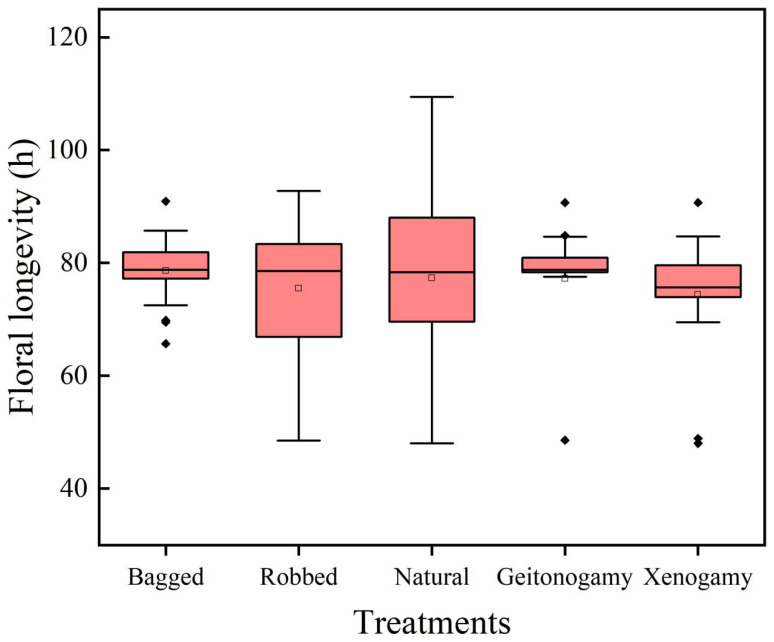
Effect of different treatments on flower longevity in *Salvia castanea*. There was no significant difference in flower lifespan among different treatments.

The longevity of the unrobbed flowers was 78.63 ± 1.28 h. No significant differences in longevity were found among the robbed flowers (75.49 ± 2.62 h, *p* = 0.441), flowers under natural conditions (77.33 ± 3.77 h, *p* = 0.753), and flowers of geitonogamy and xenogamy (77.22 ± 2.45 h, *p* = 0.292; χ^2^ = 1.392, *df* = 4, *p* = 0.846; Figure 4).

### 3.4. Amount of Pollen Needed for Sufficient Fertilization, and Deposited Pollen Counts and Their Seed Set After a Single Visit

**Figure 5:**
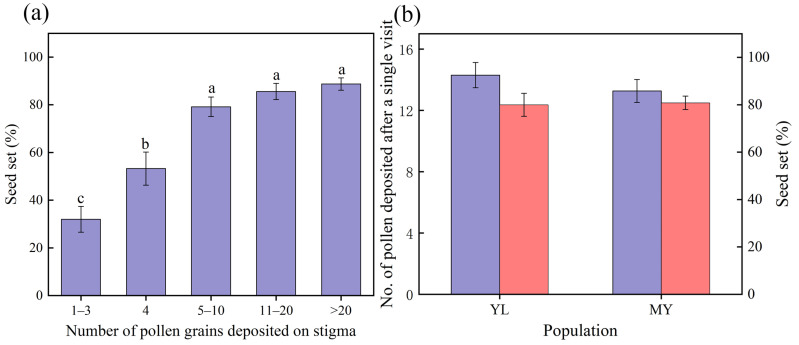
The seed set of different pollen amounts for hand pollination (**a**), the number of pollen deposited (purple) and seed set (pink) by pollinators in a single visit in two populations (**b**). Different lowercase letters indicate significant differences in the seed set of different pollen amounts for hand pollination.

Seed set significantly decreased with the different amounts of pollen for hand pollination (χ^2^ = 71.615, *df* = 4, *p* < 0.001; Figure 5a). When 1–3 and 4 pollen grains were deposited on the stigma, low seed sets of 32% and 53% were obtained, respectively. High seed sets (>80%) were found when 5–10 or more pollen grains were deposited (Figure 5a).

The number of pollen grains deposited by pollinators after a single visit was 13.27 ± 0.77 and 14.3 ± 0.82 (Figure 5b), respectively, and their seed sets were 80.00% ± 4.87% and 80.83% ± 2.81% in the two populations (Figure 5b). We found no significant differences in the number of pollen grains deposited by the pollinators (χ^2^ = 0.715, *df* = 1, *p* = 0.398; Figure 5b) or their seed sets (χ^2^ = 0.017, *df* = 1, *p* = 0.895; Figure 5b) after a single visit. These seed sets were not significantly different from those with 5–10 or more pollen grains deposited (χ^2^ = 3.578, *df* = 4, *p* = 0.466; Figure 5).

## 4. Discussion

The results of the present study revealed high spatiotemporal variations in pollinator composition and behavior in *S. castanea*. However, different levels of nectar robbing did not affect female reproductive success. We found that nectar volume was consistently replenished every evening with higher nectar concentrations and did not affect flower longevity in the robbed or unrobbed flowers. These flower traits may increase the attraction to pollinators and indirectly increase their visitation frequency. In addition, the hand-pollination results indicated that *S. castanea* required only 5–10 or more pollen grains to reach the maximum seed set. In the robbed and unrobbed populations, pollinators deposited an average of >10 pollen grains, and seed sets were not significantly different after a single visit. We next focus on the effects of spatiotemporal variations in nectar robbing on pollinators and plant reproduction in lesser ovule species, results that will drive our further understanding of plant–robber mutualisms and the ecology of nectar robbing.

### 4.1. Effects of Nectar Robbing on Pollinators and Their Spatiotemporal Variation

Our results indicated large spatiotemporal variations in the composition and behavior of pollinators of *S. castanea*. Similar results have been observed in other species of this group, such as *Salvia digitaloides* Diels (Lamiaceae), *Salvia subpalmatinervis* E. Peter (Lamiaceae), *Salvia cyclostegia* E. Peter (Lamiaceae) [41,42], *Salvia flava* G. Forrest ex Diels (Lamiaceae) [38,42], *Salvia przewalskii* Maxim. (Lamiaceae) [8,9], and *Salvia umbratica* Hance (Lamiaceae) [25]. This is consistent with the results of the ecological generalization of these species [13]. Previous research on *S. castanea* has shown that alpine environmental conditions are harsh and that there are large inter-annual differences in pollinator composition [27,28]. Thus, attracting different types of pollinators may be advantageous for *S. castanea* to ensure seed set. In sage, pollinators commonly visit several flowers on a plant in succession [13,25,27,28,36,43], and the composition of different types of pollinators may promote the deposition of mixed pollen between different *S. castanea* individuals, which may reduce the potential for inbreeding depression in plants. In addition, spatiotemporal variations in nectar robbers and pollinators may negatively affect reproduction in *S. castanea*. On the one hand, the anthers (pollen sacs) are exposed or hidden below the upper lip in *Salvia*, which can easily lead to pollen waste when insects visit the flower to obtain nectar [41,44]. On the other hand, the combination of different types of pollinators and nectar robbers may result in pollinator-related floral traits that are subjected to net selective pressure from various pollinator functional groups, ultimately leading to specialized adaptations to a particular functional group [23,45,46,47]. Because pollinators and nectar robbers share similar floral signals, those that attract pollinators are also expected to attract nectar robbers, leading to correlated selection for these floral traits. Different combinations of pollination, nectar robbing, and spatiotemporal variations are complex biological processes, and their effects on plant reproduction and evolution require further investigation.

Nectar robbing may alter relationships between plants and pollinators [17,19,48,49]. For example, this phenomenon influences the behavior of effective pollinators; in many species, bumblebees have a mixed foraging strategy (with both pollination and nectar-robbing behaviors). In the present study, *B. friseanus* acted as a pollinator and a nectar robber in 2014 and 2015. This mixed foraging strategy was also observed by Xiao et al. [25] and Ye et al. [9] in the alpine Himalayan region of Southwestern China. At high altitudes, where the climate is variable and floral resource availability may also be affected by climate change, the factors influencing changes in the behavior of effective pollinators are complex, and further research is needed. Moreover, nectar robbing reduces the proportion of effective pollinator visits. In the present study, more than 95% of the visits were nectar robbing, and the average number of visits per flower per day by effective pollinators was 0.3 and 1.3 in 2015 and 2016, respectively. Some studies have suggested that nectar robbing reduces the percentage of pollinator visits [9,50,51]. In the present study, the visit frequency of robbers only slightly reduced that of effective pollinators, indicating no significant relationship between them. Furthermore, in the unrobbed flowers, the average number of visits by effective pollinators was also found to be only 0.5 per flower per day. Previous studies on *S. castanea* have demonstrated that this is due to a shift in pollinator behavior caused by changes in weather [27] or a decrease in pollinator abundance in the area [9].

Some studies have found great variations in nectar robbing rates between years and populations, which may be due to the effects of climate or other factors (e.g., floral displays and population environments) [9,20,23,50,51]. In the present study, we also found high variations in pollinator composition and behavior between years and populations. Our previous study also found that climate is one of the factors influencing nectar robbing [27]; however, we did not find a significant correlation between flower display and nectar robbing rates (Xiao et al., unpublished). Cuevas and Rosas-Guerrero [23] studied *Salvia gesneriflora* and suggested that robbers may perceive the abundance of flowers and associate them with food availability. Previous studies have shown that *S. castanea* is ecologically generalized, with flower morphology typically adapted to multiple pollinators, and that insects may change their behavior owing to competition for nectar resources [28]. Ye et al. [9] found that community floral diversity significantly affects the flower robbing rate in *S. przewalskii*. *Salvia castanea* and *S. przewalskii* have similar ecological niche distributions, flowering periods, and community compositions, and spatiotemporal variations in nectar robbing may also be influenced by the floral resources of the community in *S. castanea*.

### 4.2. Effect of Spatiotemporal Variations in Nectar Robbing on Reproduction

The effects of nectar robbing on plant reproductive success may be mediated by nectar availability and concentration. Several studies have shown that nectar availability is strongly influenced by co-visitors and that nectar availability in robbed flowers is usually lower than that in unrobbed flowers [23,52]. In the present study, although the nectar in the unrobbed flowers was nearly twice as high as that in the robbed flowers before 14:00, it rapidly replenished to equal levels. The nectar concentrations in the robbed flowers were higher after 10:00 than those in the unrobbed flowers. Similar results have been observed in other species of *Salvia*. For example, the nectar availability in unrobbed flowers of *S. gesneriflora* is eight times higher than that in robbed flowers of this species [23], and nectar is continuously replenished in *S. przewalskii* distributed in the Himalayas of Southwestern China [8,9]. This may have two explanations. One is that water may evaporate from the robbed pore; the other is that flowers respond to nectar robbing by increasing nectar concentration to mitigate the attraction of robbing to pollinators and increase reproductive success. Another interesting finding was that nectar was more concentrated in the robbed flowers. However, in the present study, we could not determine whether it affected the visitation of different pollinators or whether it affected the reproduction of *S. castanea*. Further research should be conducted to investigate the effects of nectar from robbed and unrobbed flowers on pollinator attraction and plant reproduction. In conclusion, nectar availability may play an important role in maintaining plant reproductive success.

Robbers destroy flower structure by creating holes in the calyx or corolla tubes, which may shorten flower life or increase plant resource costs, ultimately reducing seed sets. In the present study, none of the treatments (including bagged, nectar robbing, exposure to pollinators, and hand pollination) shortened flower longevity, and similar results have been reported for *S. gesneriflora* [23]. Flowering plants often exhibit diverse flower longevity in response to environmental conditions; for example, many plants close their flowers or terminate flower longevity after pollination to save plant resources [2,25,53,54]. Conversely, the maintenance of flower longevity inevitably increases costs, which may be a selective pressure by which plants have evolved to respond to local environmental conditions (e.g., nectar robbing). In other *Salvia* species where flower longevity is correlated with pollinators or pollen deposition on the stigma, such as *S. umbratica* [25], *Salvia daiguii* Y. K. Wei & Y. B. Huang (Lamiaceae) [13,43], and *Salvia sellowiana* Benth. (Lamiaceae) [55], the flower longevity of untreated plants is significantly longer than that of plants sufficiently pollinated or exposed to pollinators. The maintenance of floral longevity may be a defense mechanism against nectar robbing, enhancing reproductive fitness by remaining attractive to pollinators in *S. castanea*.

The pollen saturation hypothesis predicts that even a significant reduction in pollinator visits to robbed plants and flowers does not affect female reproductive success when plant reproduction is not limited by pollen [22]. This is due to the fact that pollinators can deposit sufficient pollen on the stigma to fertilize all ovules after a single or a few visits [22,25]. In the present study, pollinators deposited an average of more than 10 pollen grains after a single visit, regardless of whether the flowers were robbed or not, which far exceeded the number of ovules in *Salvia* (four), and their seed sets were not significantly different from those of hand-pollinated flowers. Similar results were observed in *S. umbratica* [25]. Previous studies on *S. castanea* by Xiao et al. [27,28] showed that this species has no pollen or pollinator limitations, is self-compatible, has a low degree of autonomous self-pollination, and has no inbreeding depression. These results suggest that floral traits, breeding systems, and pollen saturation hypothesis mechanisms function together under spatiotemporal variations in nectar robbing in *S. castanea*.

In the present study, nectar robbing did not significantly affect the seed sets of *S. castanea*, despite spatiotemporal differences in the composition and behavior of pollinators and nectar robbers. We suggest that this is due to the fact that nectar availability and floral longevity maintenance mitigate the attraction of nectar robbing to pollinators. *Salvia* has only four ovules and a mixed mating system, where either autonomous self-pollination or a single visit by a pollinator easily deposits the amount of pollen needed to fertilize all ovules. These results may explain the maintenance of seed sets in the presence of spatiotemporal variations in pollinators and nectar robbers. Previous studies on *S. castanea* found that nectar robbers have significantly larger bodies than flower entrances [27]. Thus, if nectar robbing is more competitive than pollination, plants are expected to evolve floral phenotypes that are attracted to nectar robbers. Previous studies on several *Salvia* species with different floral morphology types have concluded that the diversity of floral characteristics is specific to pollinators and that plant reproduction may be influenced by several factors [28]. In the present study, nectar availability, floral longevity maintenance, and pollen saturation may have played a combined role in ensuring a constant seed set under spatiotemporal variations in nectar robbing. It is important to note that in some years with high robbing rates (e.g., 2015 in YL), pollinators averaged only 0.3 visits per flower per day. This may have a negative effect on reproduction in *S. castanea*. For example, over 80% of the seed set was recorded after a single visit by a pollinator, compared to only 60% of the population seed set. These results suggested that pollinators did not deposit sufficient pollen on the stigma of some robbed flowers. In the future, the spatiotemporal scales of the study should be increased to observe the relationship between robbing rate and seed set. In addition, we should integrate environmental and other factors with pollinator behavior, plant life history, and nectar robbing to further understand animal–plant commensalism and plant ecological evolution.

## Figures and Tables

**Table 1:** Visitation frequency of pollinators and robbers in both populations of *Salvia castanea* from 2014 to 2020. Different lowercase letters indicate significant differences between years in different populations. Values are mean ± SE. YL, Yulong Mountain; MY, Mingyin Town.

Population	YL				MY	
Year	2014	2015	2016	2020	2019	2020
Average No. of flowers observed per video per day	74 (*n* = 3 d)	52 (*n* = 3 d)	70 (*n* = 4 d)	68 (*n* = 6 d)	170 (*n* = 8 d)	71 (*n* = 6 d)
Total No. of flower visits by all insects during the observation period	548	855	4853	14268	808	3110
Total No. of flower visits by pollinators	519 (94.71%)	50 (5.85%)	217 (4.46%)	14245 (99.84%)	808 (100%)	3110 (100%)
Total No. of flower visits by robbers	29 (5.29%)	805 (94.15%)	4652 (95.54%)	23 (0.16%)	0	0
No. of visits per flower per day	2.43 ± 0.47 c	0.30 ± 0.09 d	1.35 ± 0.95 c	34.91 ± 1.50 a	0.54 ± 0.13 cd	7.26 ± 0.47 b

## Data Availability

All data will be made available on request, and additional information can be obtained from the corresponding author on reasonable request.

## Supplementary Materials

The following supporting information can be downloaded at https://www.mdpi.com/article/10.3390/plants14152266/s1, Figure S1 Effective pollination and nectar robbing behavior of S. castanea. (a) Pollinators enter the corolla tube from the flower entrance to obtain nectar, and pollen is deposited on the pistil on the back of the pollinator. (b) Nectar robbers obtain nectar from holes in the calyx and do not pollinate effectively.
