# Supplementary material for: Spatiotemporal Variations in Nectar Robbing and Its Effects on Reproduction in Salvia castanea Diels (Lamiaceae)
Source: Plants (Basel). 2025 Jul 23;14(15):2266. doi: 10.3390/plants14152266 (PMC12348264; doi:10.3390/plants14152266)
Supplement: Supplementary file 1 [file plants-14-02266-s001.zip › plants-3729216-supplementary.pdf]

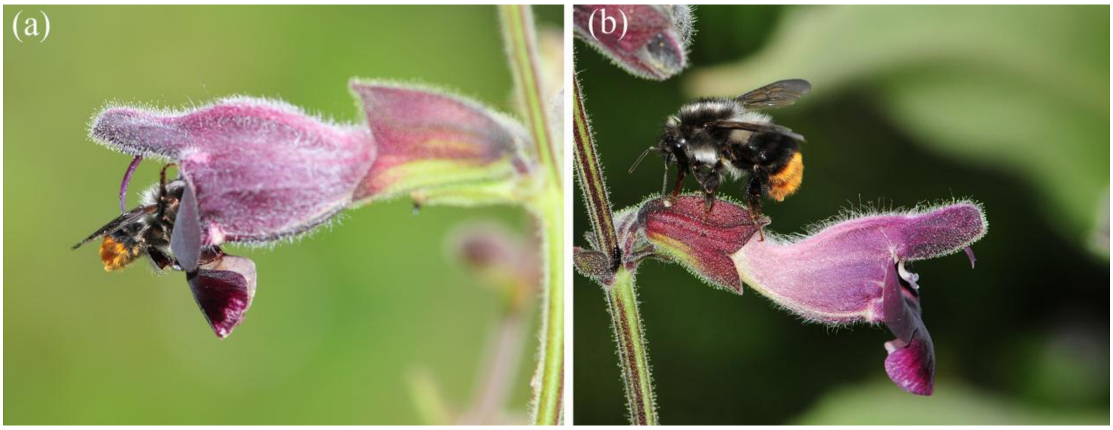

Figure S1. Effective pollination and nectar robbing behavior of S. castanea. (a) Pollinators enter the corolla tube from the flower entrance to obtain nectar, and pollen is deposited on the pistil on the back of the pollinator. (b) Nectar robbers obtain nectar from holes in the calyx and do not pollinate effectively.
